# Identification of Novel Therapeutic Targets for MAFLD Based on Bioinformatics Analysis Combined with Mendelian Randomization

**DOI:** 10.3390/ijms26073166

**Published:** 2025-03-29

**Authors:** Jialin Ren, Min Wu

**Affiliations:** 1 Institute of Translational Medicine, Medical College, Yangzhou University, Yangzhou 225100, China; 15152391113@163.com; 2 Jiangsu Key Laboratory of Integrated Traditional Chinese and Western Medicine for Prevention and Treatment of Senile Diseases, Yangzhou University, Yangzhou 225100, China

**Keywords:** MAFLD, disogenin, Mendelian randomization, drug targets, genetics

## Abstract

Metabolic-associated fatty liver disease (MAFLD) is a chronic liver condition with limited therapeutic options. To identify novel drug targets, we integrated bioinformatics, Mendelian randomization (MR), and colocalization analyses. Using the Gene Expression Omnibus (GEO) database, we identified differentially expressed genes and constructed protein–protein interaction (PPI) networks, pinpointing 10 hub genes. MR and colocalization analyses revealed that Ubiquitin-like with PHD and ring finger domains 1 (UHRF1) is causally associated with MAFLD and driven by the same causal variant locus, suggesting its potential as a therapeutic target. Molecular docking identified disogenin as a candidate small-molecule drug targeting UHRF1. Drug affinity responsive target stability (DARTS) assays confirmed direct binding between UHRF1 and disogenin. In vitro, disogenin significantly reduced UHRF1 mRNA and protein levels induced by free fatty acids (FFA) in AML12 and HepG2 cells, accompanied by decreased cellular total cholesterol (TC) and triglyceride (TG) levels. In vivo, disogenin administration alleviated hepatic lipid accumulation, inflammation, and fibrosis in methionine/choline-deficient (MCD)-diet-fed mice. This study identifies UHRF1 as a promising therapeutic target for MAFLD and validates disogenin as a potential therapeutic agent, providing a foundation for further investigation.

## 1. Introduction

Metabolic-associated fatty liver disease (MAFLD) is a prevalent chronic liver condition characterized by hepatic steatosis in the context of metabolic dysfunction, as defined by recent international consensus [1,2]. The progression of MAFLD is a dynamic process [3]. In the early stages, hepatocytes begin to accumulate lipids. As lipid accumulation increases, hepatocytes experience greater lipotoxic damage, leading to oxidative stress. This results in mitochondrial damage at the cellular level and the release of inflammatory factors, further exacerbating hepatocyte injury [4]. Metabolic-dysfunction-associated steatohepatitis (MASH) represents a more advanced stage of MAFLD. It is estimated that approximately 20% of patients with MAFLD will develop MASH [5]. Some patients with MASH may progress to cirrhosis, hepatocellular carcinoma (HCC), and ultimately, liver-disease-related mortality [6]. Notably, MASH has become the fastest-growing cause of HCC [7]. The global rise in MAFLD incidence thus represents a significant clinical challenge. Early intervention is crucial to prevent disease progression to severe stages such as MASH or HCC. Currently, the primary management strategies for MAFLD include lifestyle modifications and pharmacotherapy, largely due to the lack of specific treatments [8]. However, in clinical practice, most patients struggle to achieve weight control, and treatment outcomes are often unsatisfactory [9]. Therefore, identifying novel and specific therapeutic targets for MAFLD is essential to provide additional options for patients who do not respond adequately to lifestyle interventions or existing therapies.

Bioinformatics is a discipline that involves the collection, processing, storage, dissemination, analysis, and interpretation of biological information. As a relatively recent field, it has emerged alongside rapid advancements in life sciences and computer science. By integrating biology, computer science, and information technology, bioinformatics enables the exploration of biological mysteries hidden within large and complex datasets [10]. This field provides a systematic framework for analyzing relationships between biomarkers, drug targets, and diseases. Compared to traditional laboratory methods, bioinformatics is faster and more cost effective. It not only validates subsequent experiments but also identifies biomarkers or drug targets specific to individuals or subgroups, thereby advancing the development of personalized medicine [11]. Currently, bioinformatics is widely used in the identification of disease-related drug targets and biomarkers.

Mendelian randomization (MR) analysis is a method used to strengthen causal inference. It utilizes genetic variation as an instrumental variable (IV) to infer causal relationships between exposure and outcome [12]. Unlike observational studies, MR analyses are less susceptible to confounding by unmeasured factors due to the random and independent assortment of alleles during meiosis [13]. Additionally, genetic variation is determined at birth and remains stable over time, reducing the risk of reverse causality [14]. The binding sites of drugs and biomolecules in organisms represent potential drug targets for further investigation. Drug targets include a wide range of biological molecules, such as receptors, enzymes, ion channels, transporters, immune system components, and genes [15]. The concept of MR applied to drug targets can be understood as a form of MR involving these biological entities [16]. As a result, drug targets validated by MR are more genetically plausible and hold greater value for clinical studies.

In this study, we integrated bioinformatics and MR to identify potential drug target genes in MAFLD, offering a novel perspective on the development of targeted therapies for this condition. We anticipate that the combination of bioinformatics, MR, gene colocalization analysis, gene enrichment analysis, and protein–protein interaction network construction will provide valuable insights for developing more effective and targeted treatments.

## 2. Results

### 2.1. Differential Gene Expression Analysis in MAFLD Compared to Normal Groups

**Figure 1:**
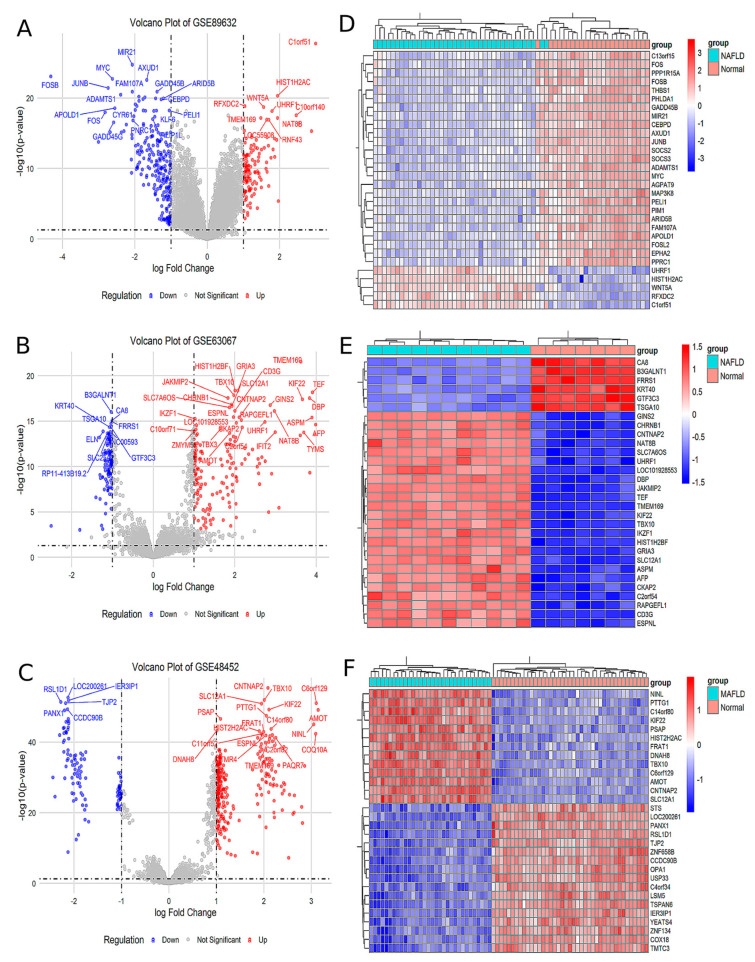
The analysis of the different GEO databases revealed the MAFLD group for DEGs compared to Normal group. (**A**) Volcano plot of the difference analysis in GSE89632. (**B**) Volcano plot of the difference analysis in GSE63067. (**C**) Volcano plot of the difference analysis in GSE48452. (**D**) Heat map of the correlation of the difference analysis in GSE89632. (**E**) Heat map of the correlation of the difference analysis in GSE63067. (**F**) Heat map of the correlation of the difference analysis in GSE48452.

Analysis of the GSE89632 dataset identified 129 upregulated and 249 downregulated genes in the MAFLD group compared to the Normal group (Figure 1A). The top 30 significantly differentially expressed genes (DEGs) were visualized in a heatmap (Figure 1D). Similarly, analysis of the GSE63067 dataset revealed 232 upregulated and 122 downregulated genes in MAFLD, with corresponding volcano plots (Figure 1B) and a heatmap of the top 30 DEGs (Figure 1E). In the GSE48452 dataset, 288 genes were upregulated and 118 were downregulated in MAFLD, as illustrated by volcano plots (Figure 1C) and a heatmap of the top 30 DEGs (Figure 1F). Comprehensive lists of DEGs are provided in Supplementary Materials, Tables S1–S3.

### 2.2. PPI Network Was Constructed in Order to Identify Hub Genes

**Figure 2:**
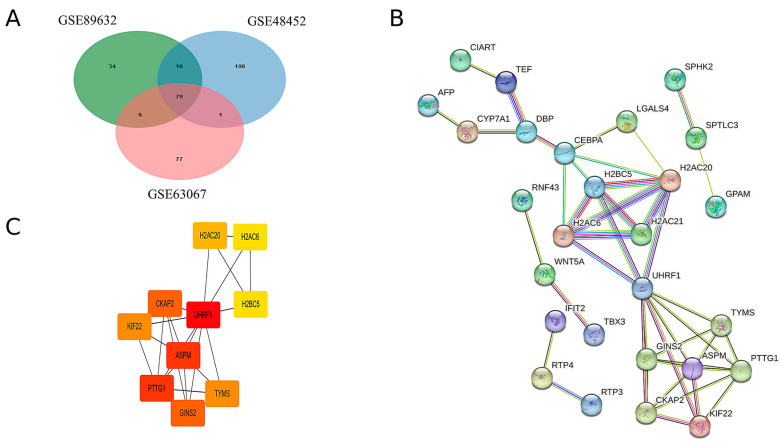
Hub genes were identified by the construction of the PPI network. (**A**) Venn diagrams of upregulated genes in the three GEO databases. (**B**) Protein interactions of upregulated genes common to MAFLD group and Normal group. (**C**) The 10 hub genes with the highest scores in the up-regulated differential genes in MAFLD versus normal tissue.

By intersecting the upregulated genes from the three GEO datasets, a total of 79 common upregulated genes were identified, as illustrated in Figure 2A. A PPI network of these common upregulated genes was constructed using Cytoscape software, version 3.10.2 and the results are presented in Figure 2B. Ten hub genes were identified using the CytoHubba plugin, as shown in Figure 2C. Detailed scores for multiple centrality measures are provided in Supplementary Materials, Table S4.

### 2.3. GO and KEGG Enrichment Analysis

**Figure 3:**
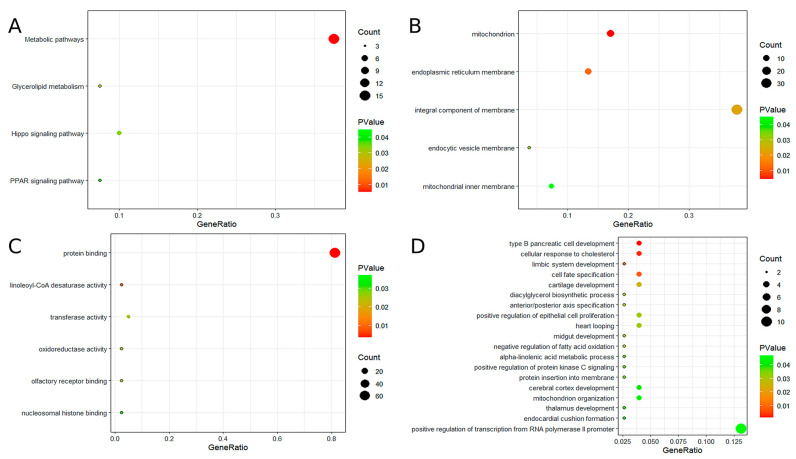
The results of GO and KEGG enrichment analysis. (**A**) KEGG enrichment analysis of candidate hub genes. (**B**) GO enrichment analysis of candidate hub genes (GO-CC). (**C**) GO enrichment analysis of candidate hub genes (GO-MF). (**D**) GO enrichment analysis of candidate hub genes (GO-BP).

To further explore the potential functions of the 10 hub genes, GO and KEGG analyses were performed. GO analysis indicated that these genes are primarily involved in the development of type B pancreatic cells, cellular response to cholesterol, mitochondrial and endoplasmic reticulum membrane functions, protein binding, linoleic acid-CoA desaturase activity, and other related biological processes. KEGG pathway analysis revealed significant enrichment in several metabolic pathways, including glycerol ester metabolism, the hippocampal signaling pathway, and the peroxisome proliferator-activated receptor (PPAR) signaling pathway. For additional details, please refer to Figure 3.

### 2.4. MR Analysis

A search of the eQTLGen database for cis-eQTL data related to the 10 hub genes yielded results for only eight genes, as H2A clustered histone 20 (H2AC20) and assembly factor for spindle microtubules (ASPM) were not included in the database. MR analysis was performed on the eight identified hub genes. SNPs located within 1 Mb of the TSS and meeting an FDR threshold of less than 0.05 were selected as IVs, resulting in the identification of 9260 SNPs. All selected SNPs satisfied the three fundamental assumptions of MR and demonstrated F-statistics greater than 10 (for detailed information, refer to Supplementary Materials, Table S3). PhenoScanner analysis confirmed that none of the selected SNPs were associated with potential confounding factors.

**Figure 4:**
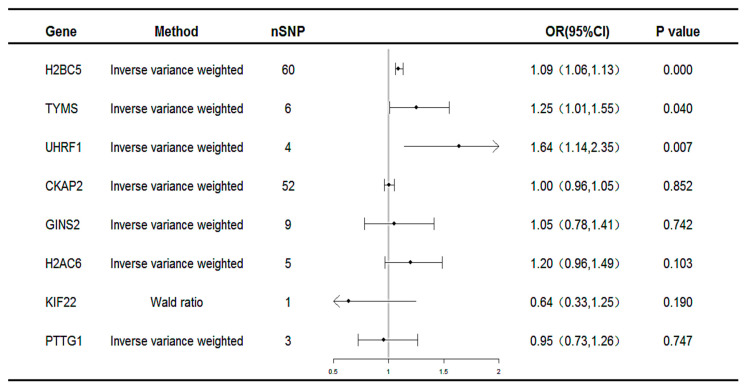
Forest plot for the causality of genes with MAFLD. CI, confidence interval; OR, odds ratio; nSNP, number of single-nucleotide polymorphisms.

Subsequent MR analyses were conducted on these co-expressed genes using the selected SNPs to assess the causal effects of each gene on the disease. The MR results identified three unique potential druggable targets: H2B clustered histone 5 (*H2BC5*), thymidylate synthetase (*TYMS*), and Ubiquitin-like with PHD and ring finger domains 1(*UHRF1*), as illustrated in Figure 4. All three genes were associated with an increased risk of MAFLD (*H2BC5*: OR 1.09, 95% CI 1.06–1.13; *TYMS*: OR 1.25, 95% CI 1.01–1.55; *UHRF1*: OR 1.64, 95% CI 1.14–2.35).

The results of the heterogeneity and pleiotropy tests for the co-expressed genes revealed *p*-values greater than 0.05, indicating no statistically significant heterogeneity or pleiotropy. Therefore, the potential impact of these factors on the results could be disregarded, as summarized in Table 1.

**Figure 5:**
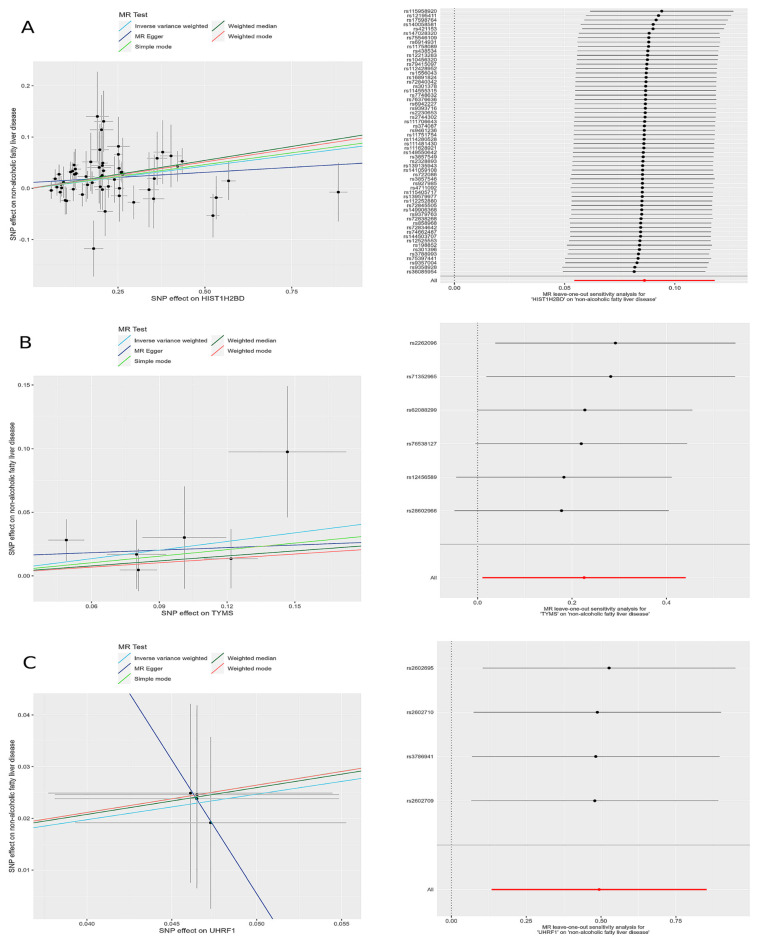
Scatter plots and leave-one-out forest maps of the 5MR models for three genes with potential causal relationships with MAFLD: (**A**) *H2BC5*, (**B**) *TYMS*, and (**C**) *UHRF1*. MR, Mendelian randomization; SNP, single-nucleotide polymorphism.

The leave-one-out sensitivity analysis revealed that the effect sizes of the included IVs were consistent with the overall effect size, confirming the robustness of the analysis. Detailed results for each gene, including scatter plots and leave-one-out sensitivity analyses, are comprehensively presented in Figure 5.

### 2.5. Colocalization Result

**Figure 6:**
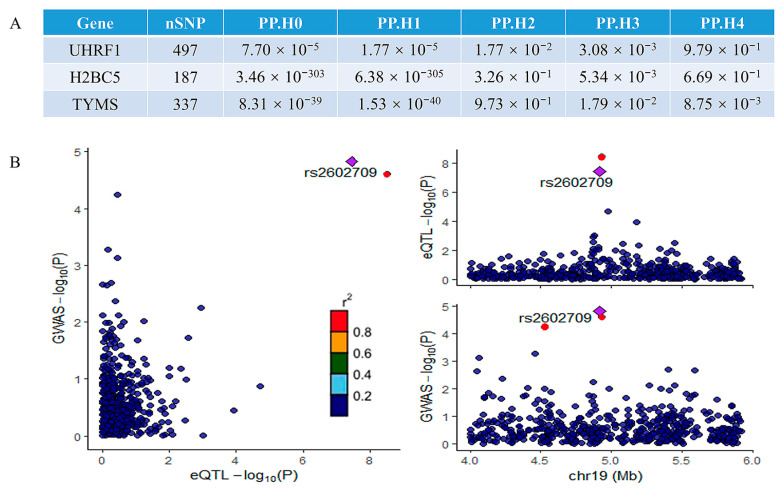
The results of the colocalization analysis: (**A**) the results of the colocalization analysis for all three potential genes; (**B**) the results of colocalization visualization of the significant gene UHRF1. The r^2^ value indicates the linkage disequilibrium (LD) between the variants and the top SNPs.

A colocalization analysis was performed to assess the overlap between the significant genes identified in the MR analysis and the MAFLD GWAS signals. This analysis identified genes colocalized with GWAS variants with high confidence (PP4 > 0.80). Among the three potentially implicated genes, only *UHRF1* demonstrated significant colocalization. The results of the colocalization analysis for all three genes are summarized in Figure 6A, while the colocalization visualization for *UHRF1* is presented in Figure 6B. The strong colocalization of *UHRF1* with MAFLD GWAS signals suggests that *UHRF1* is a key driver of genetic susceptibility to MAFLD. The colocalization analysis provides robust genetic evidence that *UHRF1* may serve as a central node in the molecular pathways underlying MAFLD pathogenesis, linking genetic risk factors to downstream pathological mechanisms and further supporting its potential as a therapeutic target. Potential reasons for the lack of colocalization in other genes include tissue-specific effects [17], regulatory complexity [18,19], pleiotropic effects [20], and other factors.

### 2.6. Identification of Disogenin as a Promising Inhibitor Targeting UHRF1

**Figure 7:**
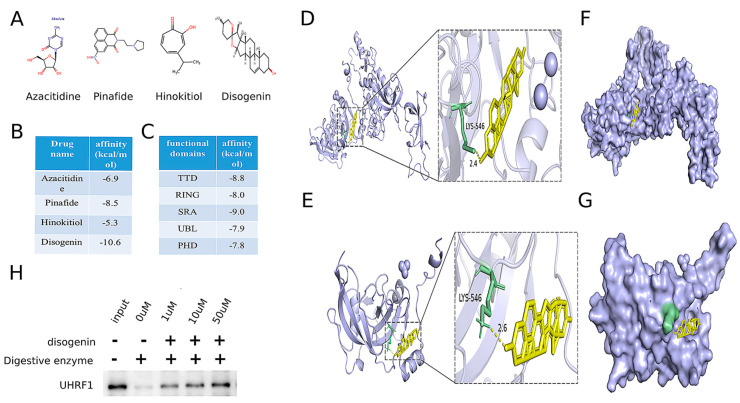
Identification of disogenin as a promising inhibitor targeting UHRF1. (**A**) The chemical structures of four small-molecule drugs that act on the MAFLD-related gene target UHRF1. (**B**) The results of these four small molecules docking with UHRF1. (**C**) The results of five domains of UHRF1 docking with five domains of UHRF1. (**D**) Molecular docking model showing the global (**left**) and local (**right**) interactions between disogenin and whole UHRF1 protein. (**E**) Molecular docking model showing the global (**left**) and local (**right**) interactions between disogenin and the SRA domain of UHRF1 protein. (**F**) The potential binding pocket at the interface of the whole UHRF1–disogenin complex was predicted. (**G**) The potential binding pocket at the interface of the UHRF1(SRA)–disogenin complex was predicted. (**H**) Western blot indicated proteolysis in the presence of disogenin in DARTS analysis.

To identify potential drug repurposing opportunities for MAFLD treatment, a comprehensive search was conducted using the ChEMBL database, DrugBank Online, and the relevant literature [21,22]. This search identified four drugs targeting the MAFLD-related gene *UHRF1*, as detailed in Table 2. The chemical structures of these four small-molecule drugs are depicted in Figure 7A. Molecular docking analyses were performed to evaluate the binding interactions between UHRF1 and each of the four small molecules. The results revealed that disogenin exhibited the lowest binding energy to UHRF1, as illustrated in Figure 7B.

UHRF1 was identified to contain five distinct domains. Molecular docking analyses were performed with disogenin using each of these five domains individually. The protein crystal structures of the UHRF1 domains were retrieved from the Protein Data Bank (PDB) (http://www.rcsb.org/pdb/home/home.do (accessed on 15 April 2024)) (PDB: 3ASL, PDB: 3FL2, PDB: 6W92, PDB: 2FAZ, and PDB: 3BI7). As shown in Figure 7C, the TDD domain exhibited the lowest binding energy. The results indicated that disogenin binds to the SRA domain of both the full-length UHRF1 protein (Figure 7D–F) and the segmental UHRF1 protein (Figure 7E–G). The consistency between the two molecular docking outcomes supports the reliability of the findings. To further validate the interaction between disogenin and UHRF1, we employed the DARTS assay. The results demonstrated that UHRF1 exhibited increased resistance to proteolysis in the presence of disogenin (Figure 7H), suggesting a direct binding interaction between disogenin and UHRF1. Based on these findings, disogenin was selected as the primary focus for further investigations.

### 2.7. Disogenin Reduced the Levels of mRNA and Protein of UHRF1 In Vitro

**Figure 8:**
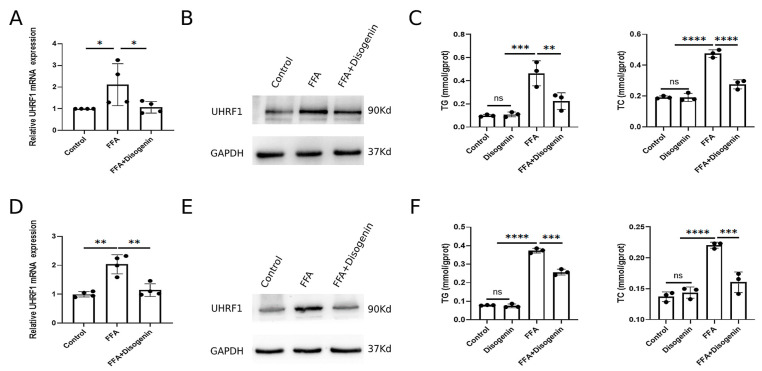
Disogenin reduced the levels of mRNA and protein of UHRF1. (**A**,**B**) The mRNA and protein levels of UHRF1 in AML12 cells. (**C**) The levels of TG and TC in AML12 cells. (**D**,**E**) The mRNA and protein levels of UHRF1 in HepG2 cells. (**F**) The levels of TG and TC in HepG2 cells. * *p* < 0.05, ** *p* < 0.01, *** *p* < 0.001, and **** *p* < 0.0001.

To investigate the effects of disogenin on UHRF1, AML12 and HepG2 cells cultured with FFA were treated with disogenin for 24 h. Subsequently, the mRNA and protein expression levels of UHRF1 were evaluated. As shown in Figure 8A,B, disogenin significantly reduced both the mRNA and protein levels of UHRF1 in FFA-treated cells. To further address the specific effects of disogenin alone, we examined UHRF1 protein expression in cells without FFA treatment (Figure S1). Notably, disogenin treatment alone did not significantly alter UHRF1 protein levels, suggesting that the regulatory effect of disogenin on UHRF1 is context-dependent and primarily manifests under FFA-induced conditions. Additionally, we measured the levels of TG and TC in both cell lines across four experimental groups. The results revealed that TG and TC levels were markedly elevated in the FFA group, while disogenin treatment significantly reduced these levels in FFA-treated cells (Figure 8C,F). In contrast, cells without FFA treatment exhibited low TG and TC levels regardless of disogenin intervention, further supporting the notion that disogenin’s lipid-lowering effects are specifically associated with FFA-induced metabolic stress.

### 2.8. Disogenin Alleviated MAFLD Development in MCD-Fed Mice

**Figure 9:**
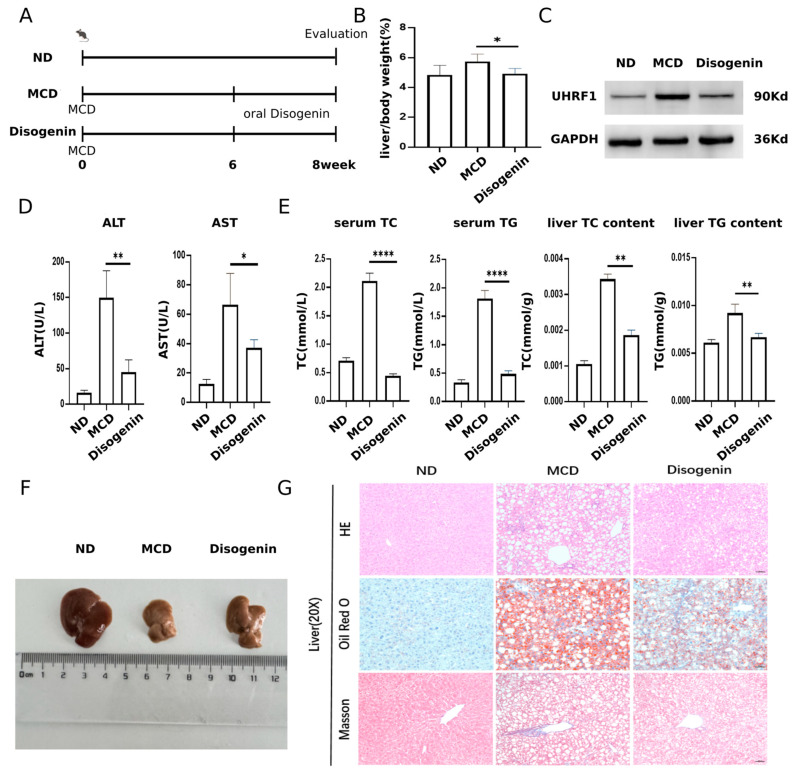
Disogenin alleviated MAFLD development in MCD-fed mice. (**A**) Schematic diagram of the experimental process in mice. (**B**) Effects of disogenin treatment on liver weight changes. (**C**) UHRF1 protein level in mice liver. (**D**) Sera of mice were isolated to detect ALT, AST. (**E**) TG and TC content of mice. (**F**) Representative images of gross liver. (**G**) H&E, Oil Red O, and Masson’s staining of liver sections (scale bar: 50 µm). * *p* < 0.05, ** *p* < 0.01 and **** *p* < 0.0001.

The experimental design for the animal studies is illustrated in Figure 9A. A significant increase in liver/body weight was observed in the MCD group compared to the ND group. However, treatment with disogenin restored liver weight to levels close to those of the control group (Figure 9B). Additionally, disogenin treatment significantly reduced the protein levels of UHRF1 (Figure 9C).

Mice fed the MCD diet exhibited a marked increase in ALT and AST activities, approximately twofold higher than those in the ND group (*p* < 0.01). In contrast, disogenin treatment significantly reduced the activities of these enzymes (*p* < 0.01) compared to the MCD group (Figure 9D). As shown in Figure 9E, the disogenin group demonstrated a substantial decrease (*p* < 0.01) in liver TC and TG levels, along with a reduction (*p* < 0.05) in serum TC and TG levels, compared to the MCD group. Although the liver size of mice in the disogenin group did not fully return to ND group levels, it showed a significant increase compared to the MCD group (Figure 9F).

Histological examinations, including H&E staining, Oil Red O staining, and Masson’s trichrome staining, revealed that the MCD diet induced pronounced macrovesicular steatosis, collagen deposition, and inflammatory responses in the liver. Scattered necrotic hepatocytes were also observed in H&E-stained sections of the MCD group. In contrast, disogenin administration significantly reduced the inflammatory area, lipid droplet size, and density compared to the MCD group (Figure 9G).

## 3. Discussion

In this study, we utilized the GEO database for bioinformatics analysis to identify differentially expressed genes, construct a PPI network, and determine 10 hub genes using Cytoscape software, version 3.10.2. Subsequently, we performed MR and colocalization analyses, along with an epidemiological investigation of genetic variation, to confirm that UHRF1 is causally associated with MAFLD and driven by the same causal variant locus. These findings suggest that UHRF1 may serve as a potential target for the development of novel therapeutic strategies for MAFLD.

UHRF1 is a critical epigenetic regulator, primarily associated with DNA methylation [23]. Studies have demonstrated that UHRF1 inhibits the reprogramming of cancer stem cells through epigenetic mechanisms, thereby suppressing HCC and ameliorating hepatocellular injury [21]. Additionally, research has shown that UHRF1 regulates the expression of genes involved in hepatic lipid metabolism, influencing fatty acid metabolism and deposition. Overexpression of UHRF1 in the livers of wild-type mice led to significant changes in glucose and lipid metabolism. Conversely, knockdown of UHRF1 in adipose tissue reduced adipocyte size and adipogenic activity, highlighting its physiological importance in glucose and lipid metabolism [24]. Glucose and lipid metabolism are central to the pathogenesis of MAFLD [25]. However, recent studies have also revealed that UHRF1 promotes adipogenesis and limits fibrosis in adipocytes [26], which appears to contrast with its role in inhibiting lipid accumulation in hepatocytes. This discrepancy may arise from differences in UHRF1’s target genes and signaling pathways across cell types. For example, in adipocytes, UHRF1 modulates adipogenesis through GPNMB and TGF-β activation, whereas in hepatocytes, it may exert its effects via adipogenesis-related pathways such as PPARγ. We acknowledge that the function of UHRF1 in MAFLD may be tissue specific and context dependent. Although these findings suggest that UHRF1 plays a significant role in the onset and progression of MAFLD, the precise mechanisms and interrelationships remain to be fully elucidated.

Disogenin, a naturally occurring compound found in various plants such as Chinese yam (Dioscorea villosa) and Rhizoma Dioscoreae nipponicae [27,28], has been shown to exert multiple beneficial effects on various biological processes, including lipid accumulation, cholesterol metabolism, fibrotic progression, inflammatory responses, and gut microbiota composition [29,30,31,32,33,34]. Previous studies have reported that disogenin enrichment in the liver can attenuate lipid accumulation, particularly in rat and mouse models, accompanied by reductions in TC and TG levels [29,30]. Additionally, disogenin upregulates the expression of hepatic genes involved in cholesterol synthesis and those encoding cytochrome P450 enzymes, thereby preventing cholestasis [31,32]. Furthermore, disogenin has been shown to downregulate the expression of inflammatory and profibrotic mediators [33,34]. Recent studies have also demonstrated that disogenin can inhibit the development of MAFLD by modulating AMP-activated protein kinase (AMPK) and liver X receptor (LXR) signaling pathways, as well as by regulating gut microbiota and related amino acid metabolism [29,35]. The findings of the present study are consistent with these experimental results.

In our study, molecular docking analysis predicted that disogenin binds to UHRF1, and subsequent in vitro experiments demonstrated that disogenin treatment significantly reduced both mRNA and protein levels of UHRF1 in FFA-induced AML12 and HepG2 cells, accompanied by a marked suppression of cellular adipogenesis. This suggests that the binding of disogenin to UHRF1 may disrupt its normal function, particularly its role in maintaining epigenetic silencing of target genes. The observed reduction in UHRF1 expression could be attributed to several potential mechanisms: firstly, disogenin binding may induce conformational changes or post-translational modifications (e.g., ubiquitination) in UHRF1, leading to its degradation via the proteasome pathway, thereby reducing protein levels [36]; secondly, disogenin might indirectly modulate UHRF1 transcription by influencing upstream regulators or signaling pathways that control its expression, such as altering the activity of transcription factors or epigenetic modifiers associated with UHRF1 gene regulation [37]; and thirdly, the binding of disogenin to UHRF1 could trigger a negative feedback loop, wherein the cell downregulates UHRF1 expression to compensate for its functional impairment [38]. Importantly, the binding of disogenin to UHRF1 and the subsequent reduction in its mRNA and protein levels are not mutually exclusive, as the initial binding event may serve as a trigger for downstream regulatory mechanisms, ultimately leading to the observed decrease in UHRF1 expression, thereby underscoring the complex interplay between small molecule–protein interactions and cellular regulatory networks. In vivo experiments further corroborated these findings, showing that disogenin treatment resulted in a significant increase in liver weight compared to the MCD group, along with a notable alleviation of lipotoxicity and cholesterol levels in MAFLD mice, as evidenced by improved liver and serum levels of TC, TG, ALT, and AST. Histological analyses, including Oil Red O staining, revealed that disogenin significantly inhibited lipid accumulation, while Masson’s trichrome and H&E staining demonstrated reduced inflammatory cell infiltration and fibrosis, collectively highlighting the therapeutic potential of disogenin in mitigating MCD-induced MAFLD. Furthermore, GO and KEGG pathway analyses elucidated the role of UHRF1 in MAFLD pathogenesis, revealing its involvement in diverse biological processes, including B-type pancreatic cell development, cellular response to cholesterol, limbic system development, glycerol metabolism, Hippo signaling pathway, and PPAR signaling pathway. The enrichment of the PPAR signaling pathway [39] supports the hypothesis that UHRF1 plays a critical role in lipid metabolism, consistent with our experimental observations of reduced hepatic steatosis following UHRF1 inhibition, while the involvement of the Hippo signaling pathway suggests that UHRF1 may regulate cellular proliferation and tissue remodeling, processes often disrupted in MAFLD [40]. Additionally, the association with glycerol metabolism and the cellular response to cholesterol underscores UHRF1’s potential impact on energy homeostasis and lipid trafficking, thereby positioning UHRF1 as a central node integrating metabolic, developmental, and signaling pathways and highlighting its potential as a therapeutic target for MAFLD. As illustrated in Figure S2, a schematic diagram summarizes the relationship among UHRF1, disogenin, and NAFLD-related pathways, offering a comprehensive overview of the proposed mechanisms.

Our study demonstrates that UHRF1 plays a significant role in the progression of MAFLD, potentially by regulating lipid metabolism and inflammatory responses. Notably, the PPAR signaling pathway is critically involved in the pathogenesis and treatment of MAFLD [39,41]. PPARs are transcriptional regulators of genes associated with a wide range of physiological functions, including lipid metabolism and inflammation, and they play a pivotal role in the pathogenesis of MAFLD and MASH [42]. The PPAR family comprises three distinct isoforms—PPARα, PPARδ/β, and PPARγ—each exhibiting unique tissue distributions and functions [43,44]. PPARα is predominantly expressed in the liver and muscle, while PPARγ is abundant in adipose tissue [45]. PPARα primarily regulates lipoprotein metabolism (LDL and HDL-C) and modulates fatty acid synthesis and oxidation, thereby maintaining lipid homeostasis during fasting and postprandial states [46]. It is well established that PPARα regulates lipid homeostasis by inhibiting sterol regulatory element-binding protein 1 (SREBP1) expression, thereby suppressing adipogenesis. In contrast, PPARγ ameliorates insulin resistance primarily through the regulation of adipokine secretion [47]. Both PPARα and PPARγ exhibit anti-inflammatory and antioxidant properties [47,48], achieved by inhibiting the expression of pro-inflammatory genes such as interleukin-1 (IL-1), interleukin-6 (IL-6), tumor necrosis factor-α (TNF-α), and transforming growth factor-β (TGF-β) [46,49]. Given UHRF1’s central role in epigenetic regulation, we hypothesize that UHRF1 may influence MAFLD progression by modulating the expression or epigenetic modification of genes within the PPAR signaling pathway. For instance, UHRF1 could regulate the transcriptional activity of PPARs or their downstream target genes through mechanisms such as DNA methylation or histone modification. Future studies should investigate the molecular mechanisms underlying the interaction between UHRF1 and the PPAR signaling pathway, which may reveal novel therapeutic targets for MAFLD. Our findings on the role of UHRF1 in MAFLD pathogenesis align with recent advancements in drug target discovery for MASH, particularly through integrated multi-omics and drug repurposing approaches. For example, transcriptomic, proteomic, and epigenomic studies have identified novel therapeutic targets for MASH, such as PNPLA3, HSD17B13, and MTARC1 [50,51,52]. These targets have been validated through functional studies and are currently being explored in clinical trials. Similarly, drug repurposing strategies have identified existing medications, including GLP-1 receptor agonists and PPAR agonists, as potential treatments for MASH by leveraging their known mechanisms of action and safety profiles [53,54]. The identification of UHRF1 as a potential therapeutic target adds to this growing list of candidates and highlights the importance of epigenetic regulation in MAFLD pathogenesis. Furthermore, the integration of multi-omics data in our study provides a comprehensive approach to target discovery that complements existing efforts in the field. GLP-1 receptor agonists have demonstrated significant efficacy in improving hepatic steatosis, inflammation, and fibrosis, as well as promoting weight loss and glycemic control. Given the complementary mechanisms of action—GLP-1 receptor agonists primarily target metabolic and inflammatory pathways, while UHRF1 inhibition may address epigenetic dysregulation and lipid metabolism—there is potential for synergistic effects in combination therapy. For instance, combining UHRF1 inhibitors with GLP-1 receptor agonists could enhance therapeutic efficacy by simultaneously targeting multiple pathological pathways in MAFLD. Future studies should explore the feasibility and benefits of such combination therapies, including their potential to improve patient outcomes and reduce disease progression. Additionally, investigating the safety and efficacy of these combinations in preclinical and clinical settings will be essential for translating these findings into practical therapeutic strategies.

Although our study suggests a potential link between UHRF1 and MAFLD, several limitations must be acknowledged. First, the precise molecular mechanisms by which UHRF1 regulates lipid metabolism and contributes to MAFLD pathogenesis remain unclear. Future studies should focus on functional validation, including UHRF1 knockdown or overexpression in hepatocyte cell lines and high-fat-diet-induced mouse models, to elucidate its role in lipid-metabolism-related pathways and interactions with other MAFLD-related genes. Second, the potential off-target effects and systemic consequences of UHRF1 inhibition warrant careful consideration. Given that UHRF1 is involved in DNA methylation, histone modification, and cell cycle regulation, its systemic inhibition may disrupt epigenetic regulation in other tissues, leading to unintended effects such as altered gene expression, genomic instability, or impaired cellular differentiation [55]. To address this, future research should explore tissue-specific delivery systems or combination therapies to minimize off-target effects while maximizing therapeutic efficacy. Third, clinical validation is essential to establish the translational potential of UHRF1 as a therapeutic target. The absence of patient-derived data in this study represents a limitation. Future work should investigate UHRF1 expression and activity in liver biopsies from MAFLD patients, correlating its levels with disease severity, progression, and treatment response. Additionally, clinical trials exploring UHRF1 inhibition—either as monotherapy or in combination with existing therapies such as GLP-1 receptor agonists or PPAR agonists—are needed to assess its safety, efficacy, and therapeutic window. Integrating multi-omics data from patient samples could further uncover the molecular mechanisms of UHRF1 in MAFLD and identify biomarkers for patient stratification. Bridging preclinical findings with clinical applications will be critical to advancing UHRF1 as a viable therapeutic target for MAFLD.

In conclusion, we have identified UHRF1 as a potential therapeutic target for MAFLD through an integrated approach combining bioinformatics, MR, and colocalization analyses. Using molecular docking, we screened the small-molecule drug disogenin as a candidate targeting UHRF1, and its therapeutic potential was subsequently validated through experimental studies. This work lays the groundwork for further detailed investigations into UHRF1 as a promising therapeutic target for MAFLD.

## 4. Materials and Methods

### 4.1. The Study’s Flowchart

**Figure 10:**
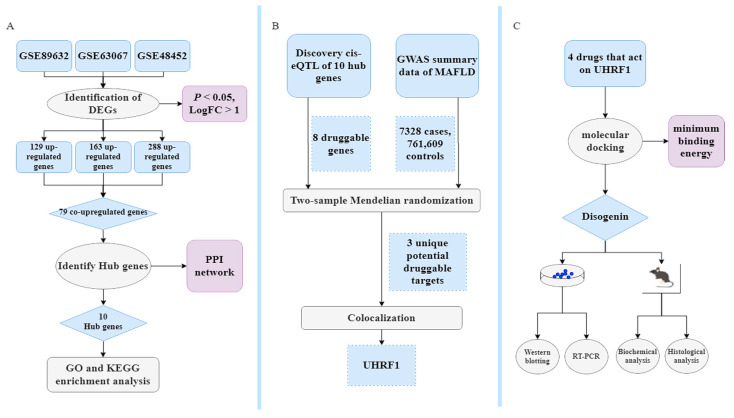
Bioinformatics, MR, identification of repurposing drugs discovery, and pharmacological analysis. (**A**) Classical Bayesian data analysis to filter differentially expressed genes (DEGs), with significance criteria set at *p* < 0.05 and LogFC > 1. (**B**) Workflow showing the MR study. The single-nucleotide polymorphism (SNP) data obtained through filtering for cis-expression quantitative trait loci (cis-eQTL) data from eQTLGen, with criteria of being located within ±1 Mb of the TSS and having a false discovery rate (FDR) < 0.05, are considered as exposure data, identifying druggable genes with strong genetic associations through the MR and colocalization analysis. (**C**) Exploring treatment drugs associated with these druggable genes that meet the inclusion criteria. The objective is to identify and validate optimal drugs that act on druggable genes. This will be achieved through molecular docking along with in vitro and vivo experimental validation.

All DNA positions are based on the human reference genome build hg19 (GRCh37). Data processing was performed using R software (version 4.3.2). The primary R packages used in this study included dplyr, limma, clusterProfiler, pheatmap, TwoSampleMR, and coloc. This MR study adhered to the guidelines outlined in the Strengthening the Reporting of Observational Studies in Epidemiology using Mendelian Randomization (STROBE-MR) statement [56,57]. The study flowchart is illustrated in Figure 10.

### 4.2. Data Collection

Gene expression datasets and clinical phenotype data matching the search terms “non-alcoholic fatty liver disease”, “*Homo sapiens*”, and “gene expression” were retrieved through microarray dataset analysis. All gene expression data and corresponding platform probe annotations are available for download from the Gene Expression Omnibus (GEO) database (https://www.ncbi.nlm.nih.gov/geo/ (accessed on 6 March 2024)).

### 4.3. Identification of DEGs

The datasets GSE89632 (39 MAFLD, 24 normal), GSE63067 (11 MAFLD, 7 normal), and GSE48452 (32 MAFLD, 41 normal) were screened for differentially expressed genes (DEGs) using R software for data management and preprocessing. The “limma” package was used for classical Bayesian analysis to identify DEGs, with a significance threshold of *p* < 0.05 and log fold change (LogFC) > 1. The “pheatmap” package was utilized to generate volcano plots and heatmaps of DEGs. Gene expression matrices and annotation files obtained from the GEO database were used for data normalization and standardization.

### 4.4. Protein–Protein Interaction (PPI) Network Construction

The online tool Search Tool for the Retrieval of Interacting Genes (STRING v12.0, https://string-db.org/ (accessed on 26 March 2024)) was used to evaluate protein–protein interaction (PPI) information. DEGs were mapped to the STRING database to assess their interaction relationships. Interactions between DEGs were screened at the protein level to construct a PPI network, including both upregulated and downregulated genes. The network was then visualized using Cytoscape software, version 3.10.2. The Cytoscape plugin cytoHubba was utilized to rank differentially expressed driver genes (DEDGs) within the PPI network based on eleven topological metrics, including degree, edge percolated component, maximal clique centrality (MCC), and six centrality measures (bottleneck, eccentricity, closeness, radiality, betweenness, and stress), all calculated using shortest-path algorithms. In this study, MCC was selected as the primary metric for identifying essential proteins in the PPI network due to its superior accuracy in predicting critical nodes, as demonstrated in previous studies [58,59]. This choice was further supported by evidence that MCC consistently outperforms other topological measures in identifying essential proteins within biological networks. The top ten hub genes were selected for further analysis.

### 4.5. Gene Ontology (GO)/Kyoto Encyclopedia of Genes and Genomes (KEGG) Enrichment Analysis

The “clusterProfiler” R package was used for GO functional annotation and KEGG pathway enrichment analysis of co-expressed genes to explore potential functional pathways and pathogenesis mechanisms. As an ontology-based tool, the “clusterProfiler” package facilitates the classification of biological terms and gene cluster enrichment analysis. A *p*-value threshold of less than 0.05 was applied as the filtering criterion in this study.

### 4.6. Expression Quantitative Trait Loci (eQTL) Analysis of Exposure Data

For the MR analysis, cis-eQTL data for blood were obtained from the eQTLGen database (www.eqtlgen.org (accessed on 10 April 2024)). The eQTLGen dataset comprises 31,684 blood and peripheral blood mononuclear cell samples, predominantly from individuals of European ancestry. The complete set of statistically significant cis-eQTL results (FDR < 0.05), updated in 2019, was downloaded. Each cis-eQTL had been validated in at least two independent cohorts [60]. Allele frequencies were derived from eQTLGen, calculated using allele counts reported across all cohorts. To assess potential horizontal pleiotropy and minimize confounding effects, we used PhenoScanner [61] (http://www.phenoscanner.medschl.cam.ac.uk/ (accessed on 14 April 2024)) to screen all eligible SNPs, excluding those associated with MAFLD-related traits (e.g., obesity and diabetes) that could act as confounders [62,63,64].

### 4.7. Determination of Outcome Data

The estimated outcome data for MAFLD were sourced from a study by Ghodsian [65] et al. (2021), published in *Cell Reports Medicine* (PMID: 34841290). This study included 778,614 samples, consisting of 7328 cases and 761,609 controls. For additional details on the dataset, please refer to the original publication [65]. The outcome data were obtained from the GWAS Catalog (https://gwas.mrcieu.ac.uk/ (accessed on 15 April 2024)). All genome-wide association study (GWAS) summary statistics used in this study are publicly available and freely downloadable. The original analysis adhered to the ethical standards established by the relevant institutional review board.

### 4.8. MR Analysis

To identify novel drug targets, cis-eQTLs of druggable genes were used as instrumental variables (IVs) in the MR analysis. First, gene symbols for all genes were obtained, and pseudogenes were excluded. Second, the transcription start site (TSS) location for each gene symbol was matched, and only SNPs within 1 Mb of the TSS with an FDR < 0.05 were included in the analysis [60,66]. Third, SNPs were grouped at r^2^ < 0.001 using European samples from the 1000 Genomes Project as IVs for MR. Finally, MR analyses were performed using the TwoSampleMR package (https://mrcieu.github.io/TwoSampleMR/ (accessed on 15 April 2024)) in R version 4.1.1. Inverse-variance weighted (IVW) was applied for IVs with more than one variant, while the Wald ratio was used for IVs with a single variant. For gene IVs containing more than two variants, sensitivity analyses were conducted using the MR-Egger and weighted median methods. Significant MR results were determined using an FDR threshold of ≤0.05.

### 4.9. Colocalization

To further investigate the observed MR associations and explore potential causal relationships, a colocalization analysis was conducted using the R package “coloc”. This analysis aimed to assess the probability of shared causal genetic variants between blood gene expression levels and MAFLD [67]. For each genomic locus, five hypotheses were tested within the Bayesian colocalization framework to evaluate the likelihood of a single variant influencing both traits. The posterior probabilities for each hypothesis were calculated as follows: PPH0, indicating no association with either trait; PPH1, indicating association with gene expression but not with MAFLD; PPH2, indicating association with MAFLD but not with gene expression; PPH3, indicating association with both traits but driven by distinct causal variants; and PPH4, indicating association with both traits due to a shared causal variant [68]. A shared causal variant suggests that the gene product may directly contribute to disease risk rather than being influenced by secondary biological processes. Genes were considered to exhibit evidence of colocalization if the posterior probability for PPH4 exceeded a threshold of 0.8, indicating a high likelihood of a shared causal mechanism [69].

### 4.10. Repurposing Drug Discovery and Molecular Docking

For potential druggable genes showing significant colocalization with MAFLD GWAS signals in the MR analysis, a gene-drug search was performed using the ChEMBL (www.ebi.ac.uk (accessed on 15 April 2024)) and DrugBank Online (go.drugbank.com (accessed on 15 April 2024)) databases, along with the relevant literature, to identify potential drugs targeting these genes. To evaluate the feasibility of subsequent experimental validation, molecular docking experiments were conducted on the selected compounds.

### 4.11. Cell Culture and Treatment

Alpha mouse liver 12 (AML12) and HepG2 cells were obtained from the Shanghai Cell Bank (Shanghai, China). Disogenin was purchased from Aoke Biology Research Co., Ltd. (Beijing, China). AML12 cells were cultured in Dulbecco’s Modified Eagle’s Medium F-12 (DMEM/F-12) supplemented with 10% FBS, 10 μg/mL insulin, 5 μg/mL transferrin, 7 ng/mL selenium, 40 ng/mL dexamethasone, and 1% antibiotic-antimycotic solution, while HepG2 cells were cultured in Dulbecco’s Modified Eagle’s Medium (DMEM) containing 10% fetal bovine serum and 1% antibiotic-antimycotic solution. Cells were seeded in 6-well plates and incubated at 37 °C in 5% CO_2_ for 24 h. The experimental groups were designed as follows: the control group was cultured in DMEM/F-12 or DMEM medium without any treatment; the FFA group was treated with free fatty acids (FFA), consisting of oleic acid and palmitic acid in a 2:1 ratio, at a final concentration of 1 mM for 24 h; the disogenin group was treated with 10 μM disogenin for 24 h after 24 h in serum-free media; and the FFA + disogenin group was first exposed to FFA (1 mM) for 24 h, followed by treatment with 10 μM disogenin for an additional 24 h.

### 4.12. Drug Affinity Responsive Target Stability (DARTS)

DARTS can recognize target proteins directly binding to small molecules without modification [70,71]. AML12 cells were seeded in 100 mm culture dishes at an appropriate density and cultured overnight in a 37 °C, 5% CO_2_ incubator. The cells were then harvested and lysed in 500 µL of RIPA buffer supplemented with protease inhibitors on ice for 30 min. The lysates were centrifuged at 12,000 rpm for 15 min at 4 °C, and the supernatants (total protein extracts) were collected and equally aliquoted into five 1.5 mL microcentrifuge tubes. Each aliquot was treated as follows: the first group received 0.1% DMSO as a control, the second group served as a negative control containing only RIPA buffer, and the third to fifth groups were treated with disogenin at concentrations of 1 μM, 10 μM, and 50 μM, respectively. All samples were incubated at 4 °C for 12 h to allow potential binding between disogenin and target proteins. After incubation, 1.25 µg of Streptomyces griseus protease was added to each tube, and the samples were digested at room temperature for 20 min. The reactions were terminated by adding 13 µL of 5 × loading buffer and boiling at 95 °C for 5 min. Subsequent operations were based on general western blot experiments [70].

### 4.13. RT-PCR Assay and Western Blotting

Total RNA was extracted from cells using the RNAiso Plus kit (Takara Bio, Beijing, China) according to the manufacturer’s instructions. RNA concentration and quality were assessed using a microspectrophotometer (Mettler Toledo UV5Nano, Greifensee, Switzerland), followed by reverse transcription to cDNA. mRNA levels of target genes were quantified using a real-time PCR system, adhering to the manufacturer’s protocols and standard procedures. The primers used for RT-PCR were as follows: UHRF1: 5′-AGGTGGTCATGCTCAACTACA-3′ (forward), 5′-CACGTTGGCGTAGAGTTCCC-3′ (reverse); GAPDH: 5′-TGCACCACCAACTGCTTAGC-3′ (forward), 5′-GGCATGGACTGTGGTCATGAG-3′ (reverse).

Cells were washed three times with cold phosphate-buffered saline (PBS) and lysed in radioimmunoprecipitation assay (RIPA) buffer. The lysate was centrifuged for 15 min, and the supernatant was collected for protein analysis. Protein levels were assessed by western blotting using the following antibodies: anti-UHRF1 (Proteintech (Rosemont, IL 60018, USA), 21402-1-AP) and anti-GAPDH (Abclonal (Woburn, MA 01801, USA), AC004).

### 4.14. Animals and Experimental Design

Five-week-old male C57BL/6J mice were obtained from the Animal Research Center of Yangzhou University (Yangzhou, China). All mice were maintained in specific pathogen-free (SPF) facilities under controlled conditions, including a 12 h light/12 h dark cycle, a temperature of 20–22 °C, and a relative humidity of 45 ± 5%. The mice had ad libitum access to standard chow and ultrapure water. The animals were fed either a normal diet (ND) or a methionine/choline-deficient diet (MCD) for 6 weeks. Subsequently, the mice were randomly divided into three groups (*n* = 6 per group) and subjected to different treatments for an additional 2 weeks: group I (ND group) received a normal diet; group II (MCD group) was fed the MCD diet; and group III (disogenin group) was administered the MCD diet supplemented with oral disogenin (30 mg/kg body weight, every other day). As illustrated in Figure 9A, liver weight was measured post-necropsy for all experimental groups at the end of the study.

All animal experiments were conducted in compliance with the institutional ethical guidelines for animal care and approved by the Ethics Committee of the Medical College of Yangzhou University.

### 4.15. Biochemical Analysis

Intracellular triglyceride (TG) and total cholesterol (TC) levels were first quantified in cell lines using commercially available assay kits. Subsequently, serum levels of glutamic-pyruvic transaminase (ALT), glutamic oxaloacetic transaminase (AST), TG, and TC were measured in animal models to comprehensively evaluate lipid metabolism. All experimental procedures were conducted in strict accordance with the manufacturer’s protocols. The assay kits were purchased from the Nanjing Jiancheng Bioengineering Institute (Nanjing, China).

### 4.16. Histological Analysis

Fixed liver tissue samples were embedded in paraffin blocks and sectioned into 4 µm thick serial slices. The tissue sections were subsequently stained with hematoxylin and eosin (H&E) (Yeasen Biotechnology Co., Ltd., Shanghai, China) to evaluate liver tissue morphology, with Oil Red O (Sigma-Aldrich Co., St. Louis, MO, USA) to visualize lipid droplet accumulation, and with Masson’s trichrome stain (G-Biosciences, St. Louis, MO, USA) to assess the extent of fibrotic changes.

## Figures and Tables

**Table 1:** Results of five MR models of known genes that reached statistical significance in IVW models and the heterogeneity and pleiotropy tests.

Gene	MR Method	*p*	Heterogeneity	Horizontal Pleiotropy
*H2BC5*	IVW	0.000	0.506	
	MR Egger	0.205	0.589	0.078
	Simple mode	0.058		
	WME	0.000		
	Weighted mode	0.001		
*TYMS*	IVW	0.040	0.590	
	MR Egger	0.853	0.482	0.644
	Simple mode	0.424		
	WME	0.331		
	Weighted mode	0.499		
*UHRF1*	IVW	0.007	0.993	
	MR Egger	0.815	0.997	0.798
	Simple mode	0.152		
	WME	0.014		
	Weighted mode	0.164		

MR, Mendelian randomization; IVW, inverse variance weighting; WME, weighted median method.

**Table 2:** Identification of a druggable MAFLD gene target and four drugs on the market for drug repurposing.

Gene	Target	Drug Name	Target ChEMBL ID	Action Type	Max Clinical Phase	Data Source
UHRF1	E3 ubiquitin-protein ligase UHRF1	Azacitidine	CHEMBL1489	Inhibitor	Approved	ChEMBL
		Pinafide	CHEMBL46874	Inhibitor	Phase 2	PubChem
		Hinokitiol	CHEMBL48310	Inhibitor	Preclinical	PMID: 37380646
		Disogenin	CHEMBL412437	Inhibitor	Preclinical	PMID: 36681316

ChEMBL, DrugBank Online, and previous publications provide all drug information.

## Data Availability

Data is contained within the article and supplementary material.

## Supplementary Materials

The following supporting information can be downloaded at: https://www.mdpi.com/article/10.3390/ijms26073166/s1.
