# Supplementary figures and images for: Identification of Novel Therapeutic Targets for MAFLD Based on Bioinformatics Analysis Combined with Mendelian Randomization
Source: Int J Mol Sci. 2025 Mar 29;26(7):3166. doi: 10.3390/ijms26073166 (PMC11989663; doi:10.3390/ijms26073166)

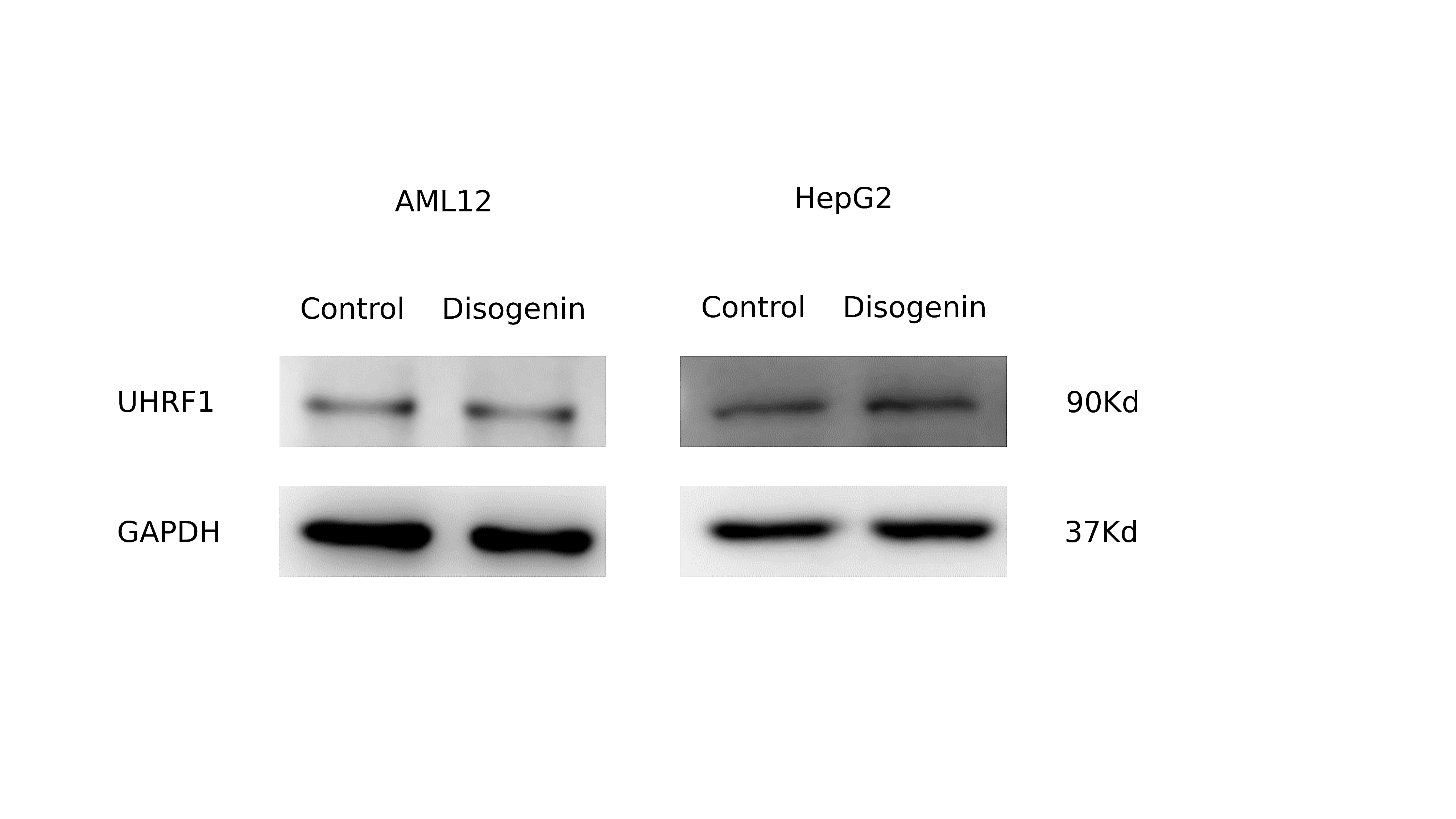

Supplement: Supplementary file 1 [file ijms-26-03166-s001.zip › Fig. S1.tiff]

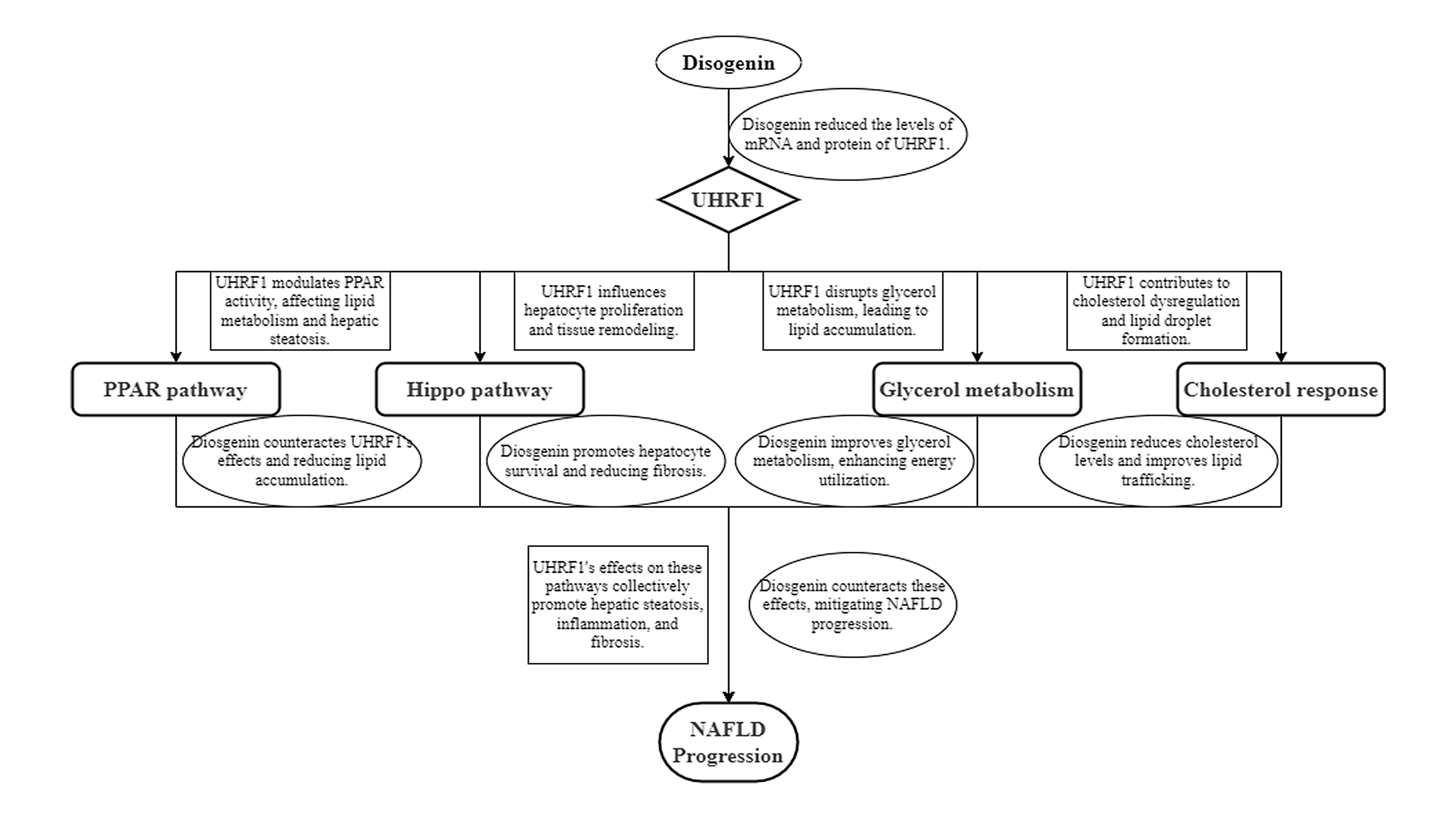

Supplement: Supplementary file 1 [file ijms-26-03166-s001.zip › Fig. S2.tiff]
